# The role of TERT promoter mutations in postoperative and preoperative diagnosis and prognosis in thyroid cancer

**DOI:** 10.1097/MD.0000000000011548

**Published:** 2018-07-20

**Authors:** Anqi Jin, Jianhao Xu, Yan Wang

**Affiliations:** a Master of Medicine in Reading, Department of Ultrasound in Medicine, Shanghai Jiao Tong University Affiliated Sixth People's Hospital, Shanghai Institute of Ultrasound in Medicine, Shanghai; b Medical College of Soochow University, Suzhou; c Department of Ultrasound in Medicine, Shanghai Jiao Tong University Affiliated Sixth People's Hospital, Shanghai Institute of Ultrasound in Medicine, Shanghai, China.

**Keywords:** FNA, TERT, thyroid cancer

## Abstract

**Background:**

Telomerase reverse transcriptase (TERT) promoter mutations have been described in different pathological types of thyroid cancers (TC). After many types of research focusing on the mutations rate in malignant tumors, the main role of TERT promoter mutations has been changed to the preoperative diagnosis and prognosis of TC, according to their high prevalence in aggressive TC.

**Methods:**

We searched Pubmed, Web of Science, Scopus, and VHL and reviewed the most common 2 mutations C288T and C250T in different types of TC, the association between them and some specific clinicopathological features, and their significance in preoperative diagnosis and prognosis of TC. we chose 38 studies into our qualitative research. We also chose 22 studies to do a meta-analysis on this subject.

**Results:**

The overall rate of these mutations in different types of TC was 10.0%, with 86.1% C228T mutation, 12% C250T mutation, and 2.1% other type mutations. The rate increases significantly as the TC become more aggressive, and reaches 56.8% in anaplastic thyroid cancer (ATC). Statistically meaningful association is found between TERT promoter mutations and older age, larger tumor size, extrathyroidal extension, lymph node metastasis, distance metastasis, advanced TNM stage, recurrence, and BRAF V600E mutation. Some studies concentrating on DNA sequencing based on fine needle aspiration biopsy (FNAB) also proved their significance in preoperative stage, with 7% to 16.5% sensitivity.

**Conclusions:**

TERT promoter mutations were likely to occur in BRAF V600E positive TC. Patients with these 2 combined mutations were more likely to have a poor prognosis and outcome. TERT promoter mutations is an essential part of the ThySeq gene panel. The mechanism of how they influence the appearance and development of TC has not been expounded. The next study direction may be the mechanism exploration and the further study to prove their significance in preoperative diagnosis.

## Abstract 2

Supplemental Digital Content is available in the text

## 1 Introduction

Telomeres are nucleoprotein complexes, consisting of several short nontranscribed DNA sequence TTAGGG and protein.^[1,2]^ They protect the ends of chromosomes from shortening with cell division by losing their DNA sequence.^[3]^ Each time the cell divides, the telomeres become shorter. When the length of telomeres reaches a critical point, cells cannot be divided any more and become senescence.^[4]^ Telomerase is an enzyme which can add telomeric DNA TTAGGG to the end of chromosomes to provide telomeres from shortening.^[5]^ It consists of human telomerase reverse transcriptase (hTERT), human telomerase RNA component (*TERT*) and human telomerase associated protein (hTP1).^[6]^ Telomerase RNA Component (TERC) serves as the template in replication, while *TERT* is responsible for catalyzing the addition of the DNA fragments TTAGGG.^[7]^ In general, there is a low expression of telomerase activity in most normal tissues and benign tumors, but a high expression in malignant tumors, related with the malignant tumor cells’ durable division ability.^[8]^ So that, the *TERT* mutations become an important research focus in the occurrence mechanism of cancer and was firstly described in the thyroid in 2013.^[9]^*TERT* gene, located on chromosome 5, consists of 16 exons and 15 introns spanning 35 kb.^[7]^ The core promoter of *TERT* including 330 bp upstream of the translation start site, is the most commonly described mutations region in recent studies. The *TERT* promoter mutations mostly occurred in 2 hotspots, 1 295 228 C > T and 1 295 250 C > T (C228T and C250T).^[8]^ These 2 mutations correlate with initiation codon ATG and tumor malignance and aggressiveness as reported. Therefore, this review is to examine and conclude these data on *TERT* promoter mutations in the thyroid.

## 2 Methods

We searched Pubmed, Web of Science, Scopus, and VHL up to 2018/2/1 with search strategy: (telomerase OR TERT OR “TERT protein, human” [Supplementary Concept]) AND (“Thyroid Neoplasms”[Mesh] OR Thyroid Neoplasm OR Thyroid Carcinoma OR Cancer of Thyroid OR Thyroid Cancer OR Cancer of the Thyroid OR Thyroid Adenoma). We found 77 records in total after removing the duplicates. Two independent investigators (AJ and JX) selected the studies in which samples are nonselected of thyroid tumors and the studies whose aims were searching the prevalence of *TERT* promoter mutations in TC and/or their association with clinicopathological features and prognosis of TC. After removing all unqualified records and some review papers, we chose 38 studies into our qualitative research. We also chose 22 studies to do a meta-analysis on this subject including 19 using paraffin embedding tissue (based on recent 2years’ studies; Table 1) and 3 using fine-needle aspiration samples. All analyses were based on previous published studies, thus no ethical approval and patient consent are required.

**Table 1:**
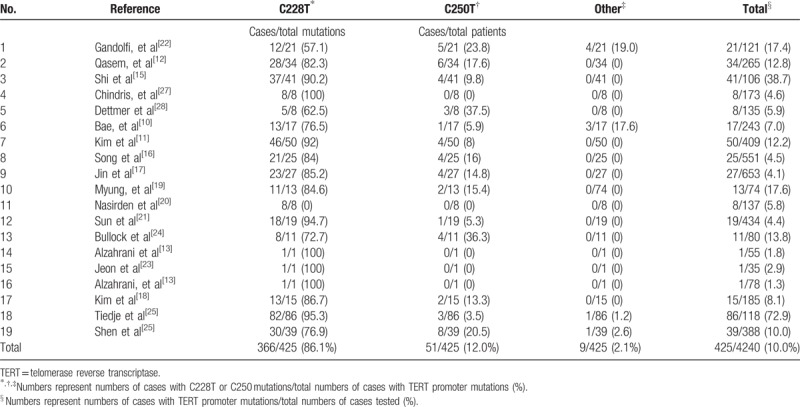
Frequency of TERT promoter mutations in TC.

## 3 Results

### 3.1 TERT promoter mutations in thyroid cancer

**Figure 1:**
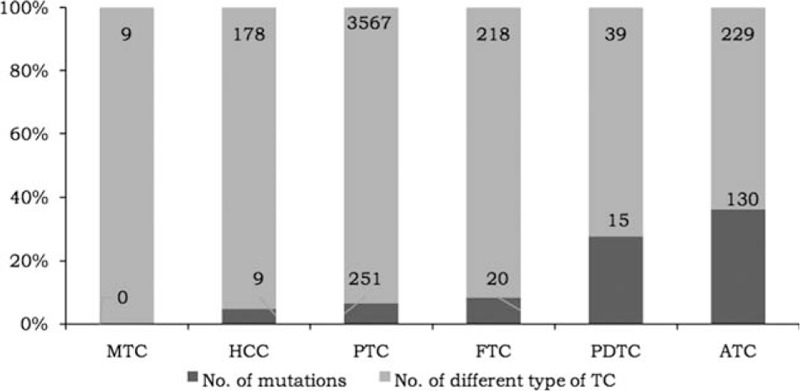
Number of cases with *TERT* promoter mutations in different types of TC. ATC = anaplastic thyroid cancer, FTC = follicular thyroid carcinoma, HCC = Hurthle cell thyroid cancer, MTC = medullary thyroid carcinoma, PDTC = poorly differentiated thyroid carcinoma, PTC = papillary thyroid cancer.

We have analyzed 19 studies that described the frequency of *TERT* promoter mutations in different types of TC.^[10–28]^ These studies included 4240 samples, and 425 *TERT* promoter mutations (10.0%) were found in total (Table 1). *C228T* mutations occurred in 366 samples (86.1%), while *C250T* mutations occurred in 51 samples (12%), other type of mutations occurred in 9 samples (2.1%). The 2 mutations were mutually exclusive except one sample, which has *C228T* mutations and *C250T* mutations at the same time (Table 1). The positive rate of mutations has much difference in different types of TC (Fig. 1). The frequency of *TERT* promoter mutations increases as the tumors become less differentiated. The percentage of these 2 mutations respectively were 0%, 5.1%, 7.0%, 9.2%, 38.5%, and 56.8% in medullary thyroid carcinoma (MTC), Hurthel cell thyroid cancer (HCC), papillary thyroid carcinoma (PTC), follicular thyroid carcinoma (FTC), poorly differentiated thyroid cancer (PDTC), and anaplastic thyroid cancer (ATC) (Fig. 1). No *TERT* promoter mutations were found in MTCs. The prevalence of *TERT* promoter mutations in HCC, PTC, and FTC were about the same, and there was no statistical difference between them (*P* > 1). The rate of mutations in PDTC and ATC was much higher than it in other subtype of TC. Six studies tested 268 cases of *TERT* promoter mutations in poorly differentiated and anaplastic thyroid cancer (PDTC and ATC) in total.^[10–12,14,25]^ 145 (54.1%) *C228T* and *C250T* mutations were found in these cases (Fig. 1). It is note worthy that the high mutations proportion in these 2 specific TC subtypes may indicate that *TERT* promoter mutations are related to TC which were more malignant. In addition, Alzahrani et al^[13,14]^ successively tested 55 pediatric TCs and 78 pediatric differentiated TCs, and only 1 case with these mutations was found, respectively (1.8% and 1.3%, respectively). So that, in pediatric thyroid cancers, the value of *TERT* promoter mutations as a single measure to identify malignant tumors from benign ones is also limited.

### 3.2 Association of *TERT* promoter mutations with clinicopathological features and adverse outcomes of thyroid cancer

As mentioned above, *TERT* promoter mutations perhaps related to poor prognosis and outcomes of TC, according to its high prevalence in PDTC and ATC. Several studies paid attention to this phenomenon and tried to analyze their association with some specific clinicopathological features. Overall, the features included age, gender, tumor size, extrathyroidal extension, lymph node metastasis, multifocality, distance metastasis, TNM stage, recurrence, and the relationship with *BRAF* mutation. We selected 9 recent studies, 2562 samples in total, including 1499 PTCs and 1063 mix pathological TC.^[10,11,16,17,19–22,24]^Table 2 summarized the brief information of each research above, and we will describe our findings in the next section in detail. The funnel plots of each separate analysis can be found in the Supplemental file. 1–10.

**Table 2:**
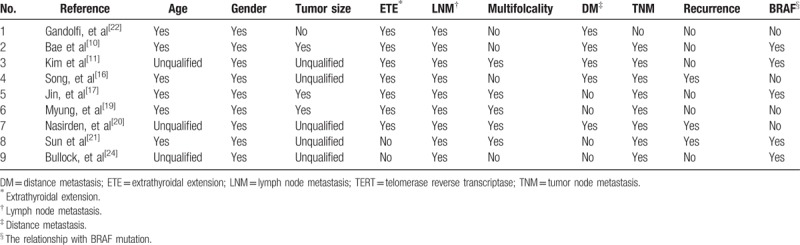
Summary of researches that reported the association between TERT promoter mutation and clinicopathological features, outcome, BRAF mutation of TC.

### 3.3 Age

**Figure 2:**
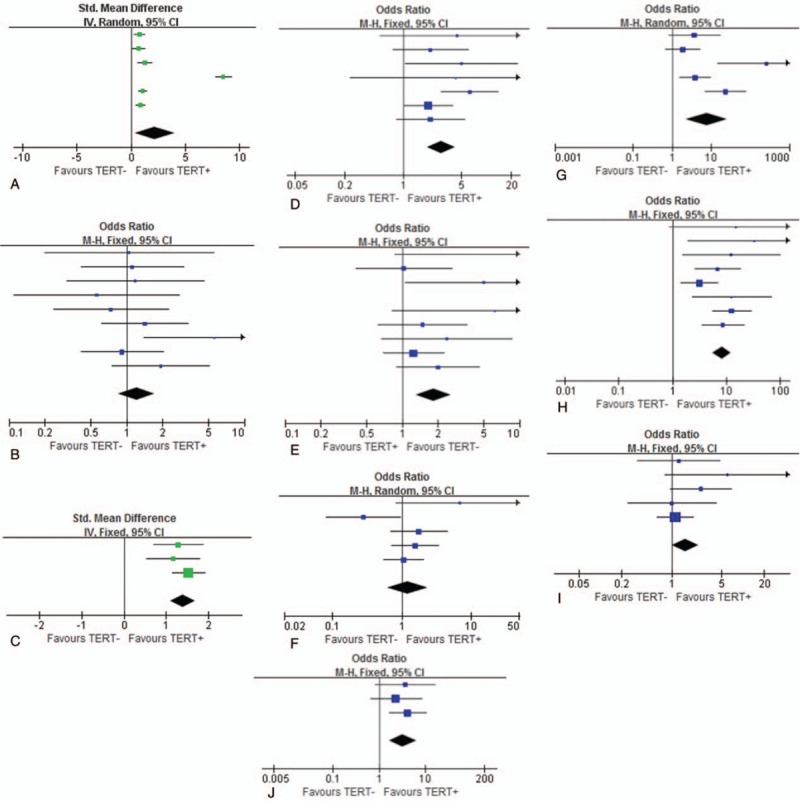
The association between *TERT* promoter mutation and clinicopathological features and adverse outcomes. Forest plot showing the association between TERT promoter mutations and clinicopathological features and adverse outcomes. (A–J) represents age; gender; tumor size; extrathyroidal extension; lymph node metastasis; multifocality; distance metastasis; TNM stage; *BRAF V600E* mutation; recurrence. The test of heterogeneity is at the left lower corner of each graph. A fixed-effect model was used when I2 was < 25% and *P* value > .05. In other situations, a random-effect model was used for the meta-analysis.

Pooled data from 6 studies^[10,16,17,19,21,22]^ (heterogeneity: Tau:5.05, Chi^2^ = 382.34, *I*^2^ = 99%, *Z* = 2.36, *P* < .01, Random efforts model)showed us that *TERT* promoter mutations tended to present in older patients (Fig. 2). In total, the patient age is 57.41 ± 8.95 vs 44.40 ± 10.32 years in *TERT* promoter mutation-positive vs mutation –negative patients (*P = *.02). In one research did by Sun in 2016,^[21]^ the patient age was 52.79 ± 4.74 vs 40.4 ± 1.11 years in positive group vs negative group. This data have a major heterogeneity from other studies (Fig. 2).

### 3.4 Gender

All 9 studies analyzed the association between *TERT* promoter mutations and patients gender ^[10,11,16,17,19–22,24]^ (heterogeneity: Chi^2^ = 7.99, *I*^2^ = 0%, *Z* = 1.05, *P = *.29, Fixed efforts model). In the meta-analysis, we did not find any statistic difference between them (Fig. 2). However, in Bullock et al.'s research^[24]^ in 2016, he found that these mutations tended to happen in female more than in male (*P = *.01). This might be explained by the lack of male patients (14 male patients in total 80 patients) in his research.

### 3.5 Tumor size

We chose 3 studies^[10,17,19]^ (heterogeneity: Chi^2^ = 1.03, *I*^2^ = 0%, *Z* = 9.38, *P* < .01, Fixed efforts model) to do the meta-analysis and found that *TERT* promoter mutations were relevant to larger tumor size (Fig. 2). In other 5 studies,^[11,16,20,21,24]^ whose data about tumor size was unqualified, *TERT* promoter mutations were also found to have high prevalence in larger thyroid tumors (*P < *.01 in all 5 studies, respectively).

### 3.6 Extrathyroidal extension

Seven studies provided the data of association between *TERT* promoter mutations and extrathyroid extension ^[10,11,16,17,19,20,22]^ (heterogeneity: Chi^2^ = 6.47, *I*^2^ = 7%, *Z* = 5.23, *P < *.01, Fixed efforts model). As we can see in Figure 2, patients with these 2 mutations were more likely to have thyroid tumors extending out of thyroid. It also reminds us that this can be a signal implying the significance of *TERT* promoter mutations of the prognosis of TC.

### 3.7 Lymph node metastasis

We included all nine studies in the meta-analysis ^[10,11,16,17,19–22,24]^ (heterogeneity: Chi^2^ = 8.58, *I*^2^ = 18%, *Z* = 3.55, *P < *.01, fixed efforts model), finding that *TERT* promoter mutations can increase the risk of lymph node metastasis with the odds ratio (OR), 1.85; 95% CI; [1.32–2.59] *P = *.0004 (Fig. 2). However, in respective studies, only 3 of them^[10,11,17]^ indicated a positive relationship between the mutations and LNM (*P < *.05). Bae et al^[10]^ and Myung et al^[19]^ provided the data of lateral lymph node metastasis. Bae et al. found the mutations were relevant to lateral lymph node metastasis (*P < *.0001) while Myung et al did not.

### 3.8 Multifocality

We collected data from 5 studies ^[11,17,19–21]^ (heterogeneity: Tau^2^ = 0.31,Chi^2^ = 9.16, *I*^2^ = 56%, *Z* = 0.57, *P* = 4.57, random efforts model), and 3 of them^[19–21]^ tells that multifocal thyroid tumors are more likely to occur in *TERT* promoter mutation-positive patients (*P < *.05). In some early studies, authors used to use vascular invasion as a single indicator of invasion,^[29,30]^ but more studies tended to choose multifocality in recent years. In pooled data, we did not see significant difference between the mutation-positive and mutation-negative group (95% CI; [0.62, 2.39] *P = *.57; Fig. 2).

### 3.9 Distance metastasis

Distance metastasis has always been an important evaluation index of prognosis. The most common distance metastasis caused by thyroid cancer were osseous metastasis and pulmonary metastasis.^[31]^ Five studies brought this clinicopathological feature into their studies^[10,11,16,20,22]^ (heterogeneity: Tau = 1.42, Chi^2^ = 17.29, *I*^2^ = 78%, *Z* = 3.25, *P < *.01, random efforts model), and 4 of them^[10,11,16,22]^ made a conclusion that *TERT* promoter mutations were relevant to it. According to the result of meta-analysis (Fig. 2), *TERT* promoter mutations were relevant to the distance metastasis with the odds ratio = 7.67 (95%CI, [2.24, 26.25] *P = *.001.

### 3.10 TNM stage

The TNM stage is a briefly conclusive clinicopatholigical feature describing the malignance of the tumor. In most studies, authors defined TNM I-II as the advanced TNM stage, but Bullock et al^[24]^ included TNM I-III all as the advanced TNM stage. Eight studies ^[10,11,16,17,19–21,24]^ (heterogeneity: Chi^2^ = 8.15, *I*^2^ = 14%, *Z* = 10.32, *P < *.01, fixed efforts model) reported a significant association between *TERT* promoter mutations and the advanced TNM stage. In the meta-analysis of them, we got a similar conclusion that these mutations were relevant to the advanced TNM stage. The heterogeneity of the analysis was small (*P = *.32), and the odds ratio was 8.24 (95%CI, [5.52, 12.30] *P < *.001]; Fig. 2).

### 3.11 Recurrence

Three studies ^[16,20,21]^ (heterogeneity: Chi^2^ = 0.55, *I*^2^ = 0%, *Z* = 3.32, *P < *.01, fixed efforts model) provided detailed data on recurrence in the mutation-positive and mutation-negative group. In the pooled data, we found thyroid cancer were more likely to recur in patients with *TERT* promoter mutations according to the meta-analysis (Fig. 2). Furthermore, 5 studies^[11,16,20,22,24]^ followed-up TC patients for years, and they all reported that these mutations would decrease the proportion of TC patients’ survival or disease-free survival.

### 3.12 *BRAF V600E* mutation

*BRAF V600E* mutation was a classic mutation harbored in thyroid cancers.^[32]^ Many articles have reported its significance in diagnosis and prognosis of TC.^[33–36]^ In our review of recent studies, several of them analyzed the relationship of it and *TERT* promoter mutations directly,^[10,11,17,21,24]^ and several analyzed the combined positive rate of it and *TERT* promoter mutations.^[10,11,16,20,22,24]^ In some respective studies,^[10,11,17,24]^*BRAF* mutation was found not relevant to *TERT* promoter mutations, however, in the meta-analysis, we got a different finding. According to the pooled data of 5 studies (heterogeneity: Chi^2^ = 4.40, *I*^2^ = 9%, *Z* = 2.09, *P = *.35, fixed efforts model), we found that *TERT* promoter mutations were likely to occur in *BRAF V600E*-positive TC (Fig. 2). Several studies tested the combined positive rate of *BRAF* mutation and *TERT* promoter mutations, and found all that patients with these 2 combined mutations were more likely to have a poor prognosis (distance metastasis, lymph node metastasis and so on) and outcomes (recurrence, TC-related mortality).^[11,16,20,22]^ Xing found that coexisting BRAF V600E and TERT C228T mutations were more commonly associated with a poor outcome such as recurrence of PTC than they were individually.^[30]^

### 3.13 The significance of *TERT* promoter mutations in preoperative diagnosis

As we can tell the importance of *TERT* promoter mutations in the diagnosis and prognosis of TC,^[9]^ the next step would be applying it in the preoperative stage. Ultrasound guided fine-needle aspiration has become the most commonly using method, which could achieve suspicious thyroid tumor cells perioperatively.^[37,38]^ Liu and Xing^[39]^ tested *TERT* promoter mutations along with *BRAF* V600E mutation by direct DNA sequencing on 308 FNAB specimens in 2014. In these studies, the authors found a 100% diagnosis specificity (9/9) and 7% sensitivity (9/129). The sensitivity was lower than it in post-operational test. This might result from the different quantity of cell in aspiration samples and paraffin embedding tissue.^[39]^ When using the combined rate with *BRAF* V600E mutation, the sensitivity increased to 38.0% (49/129). They also described the situation of these nine patients with *TERT* promoter mutations in detail and found about 80% of their thyroid nodules were thyroid cancers with aggressive clinicopathological behaviors, such as extrathyroidal invasion, lymph node metastases, distant metastases, disease recurrence, or patient death. The same year, Nikiforov et al^[40]^ test 143 FNA samples with a cytological diagnosis of follicular or oncocytic (Hurthle cell) neoplasm/suspicious for a follicular or oncocytic (Hurthle cell) neoplasm (FN/SFN) from patients using the targeted ThyroSeq v2 NGS panel. The total sensitivity was 90% and the specificity was 93%. Four *TERT* promoter mutations-positive samples were found, and 39 malignant nodules were found. These 4 samples were all thyroid cancers proved by surgical outcomes, with 10.3% sensitivity and 100% specificity. The high sensitivity may result from the selecting of only FN/SFN samples. Later in 2016, Lee et al^[41]^ reported a similar research carried on 242 TC patients and analyzed the association between *TERT C228T* mutation and clinicopatholigical features of PTC patients especially. They reported 16.5% sensitivity (39/236) and a 97.5% specificity (39/40) with one mutation harbored in a thyroid follicular adenoma. He also described that among all the clinicopathological features, the *TERT C228T* mutation was found associated with recurrence (*P = *.03) only. Coexistence of *TERT C228T* and *BRAF V600E* mutations was found in 13.0% of PTCs and was significantly associated with older age and advanced stage. We did a meta-analysis on these 3 studies and found the total sensitivity and specificity was 11.41% and 99.66%, both higher than them in the test using postoperative samples. In 2018 Nikiforova et al^[42]^ improved the ThyroSeq v2 NGS panel into a ThyroSeq v3 NGS panel. They used 175 FNA samples of indeterminate cytology (Bethesda III, n 5 84; Bethesda IV, n 5 74; and Bethesda V, n 5 17). The latest panel included 112 genes (including 2 main *TERT* promoter mutations) DNA- and RNA-based, targeted NGS assay that tests for 5 classes of genetic alterations: point mutations, insertions/deletions, gene fusions, copy number alterations, and abnormal gene expression. The total sensitivity was 98%, specificity was 81.1% (Them did not give the separate sensitivity and specificity of each gene mutation.). Such high sensitivity and specificity shows the excellent prospects of gene panel test in preoperative diagnosis of TC. Crescenzi et al^[43]^ tried to use core needle biopsy (CNB) to minimize the risk of false negatives due to lack of cell quantity in FNA. Since the total mutation rate of *TERT* promoter is comparatively low, the best way to apply them in preoperative diagnosis should consider joint gene test. Despite the next-generation sequencing using ThySeq gene panel, we thought picking out several meaningful targeted gene to create gene panel could be a more economical and suitable for different regions. So that the further confirmation of the significance and operability of *TERT* promoter mutations in preoperative diagnosis is valuable.

## 4 Conclusions

Since TERT promoter mutations were first found in thyroid cancer, it has become a hotspot of TC research. After exploring their value of the diagnosis of thyroid nodules, more studies have been done focusing on their significance in the prognosis of TC. The specialness of *TERT* promoter mutations was their high specificity in TC diagnosis and high prevalence in aggressive TC. *TERT* promoter mutations were likely to occur in *BRAF V600E*-positive TC. Patients with these 2 combined mutations were more likely to have a poor prognosis and outcome. However, more studies should be done to confirm their effect in preoperative diagnosis. Furthermore, the mechanism of how these mutations influence the happen or development of thyroid cancer has not been discovered. The special treatment such as gene drug or therapy targeting on *TERT* promoter mutations could become a new research area in the future.

## Author contributions

**Conceptualization:** Anqi Jin, Yan Wang.

**Data curation:** Anqi Jin, Jianhao Xu.

**Formal analysis:** Anqi Jin, Jianhao Xu.

**Methodology:** Anqi Jin.

**Resources:** Anqi Jin.

**Software:** Jianhao Xu.

**Writing – original draft:** Anqi Jin.

**Writing – review & editing:** Yan Wang.

## Supplementary Material

Supplemental Digital Content
