# Supplementary material for: The role of TERT promoter mutations in postoperative and preoperative diagnosis and prognosis in thyroid cancer
Source: Medicine (Baltimore). 2018 Jul 20;97(29):e11548. doi: 10.1097/MD.0000000000011548 (PMC6086515; doi:10.1097/MD.0000000000011548)
Supplement: Supplemental Digital Content [file medi-97-e11548-s001.pdf]

Supplement files. 1-10 The funnel plots of each separate meta-analysis.

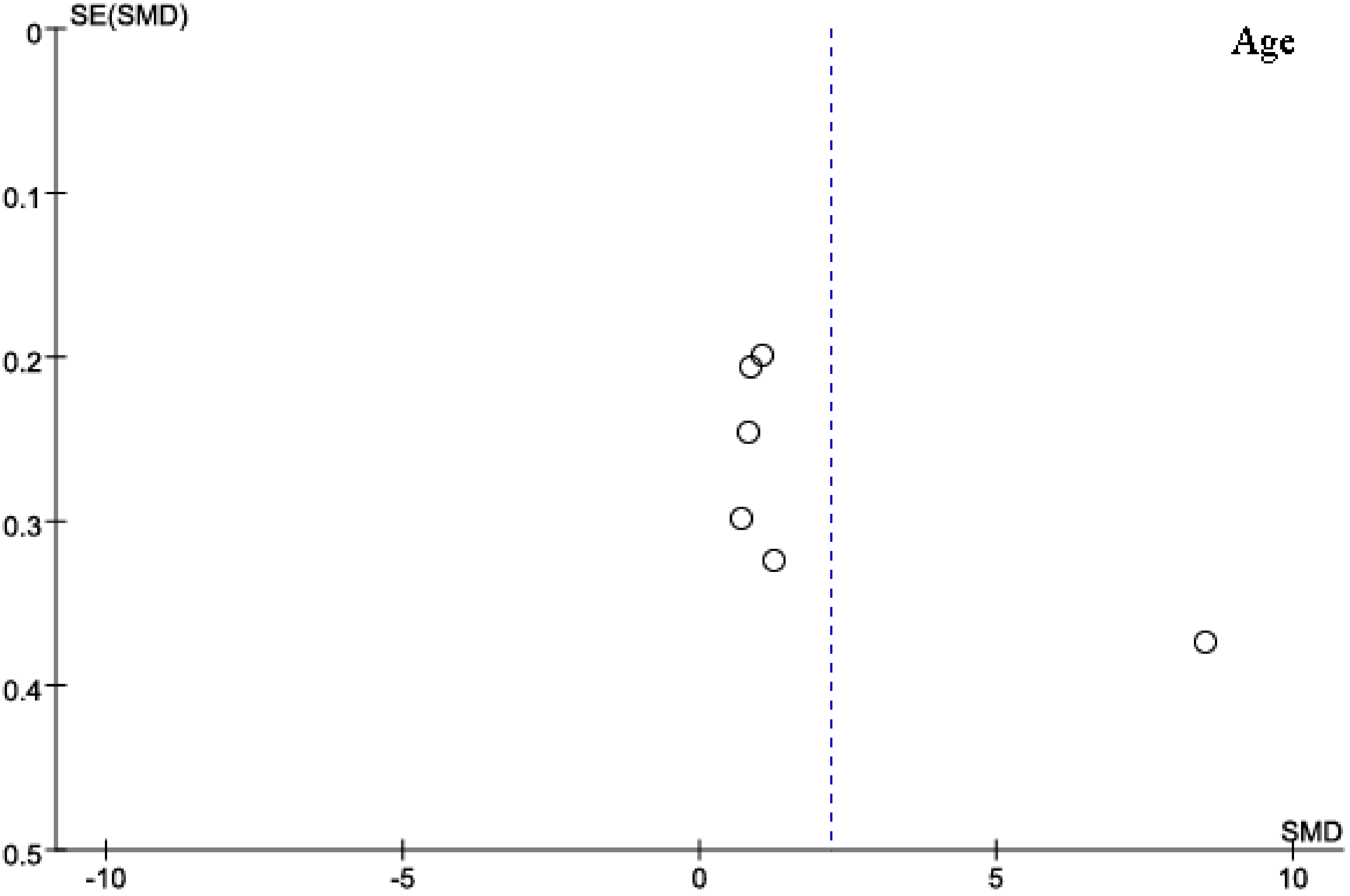
SE(SMD)
Age
0
0.1
0.2
0.3
0.4
0.5
SMD
-10
-5
0
5
10

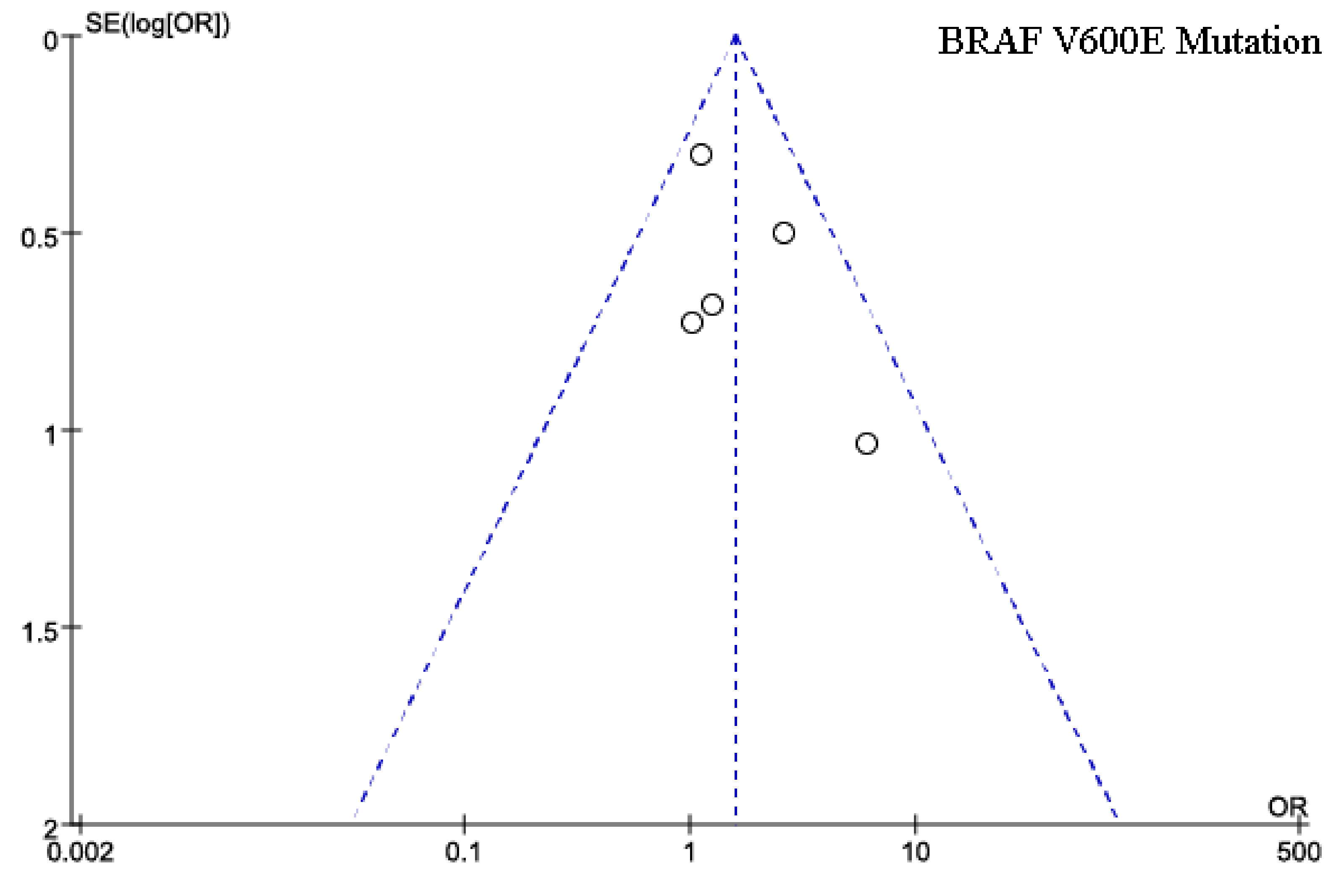
SE(log[OR])
BRAF V600E Mutation
0
0.5
1
1.5
2
OR
0.002
0.1
1
10
500

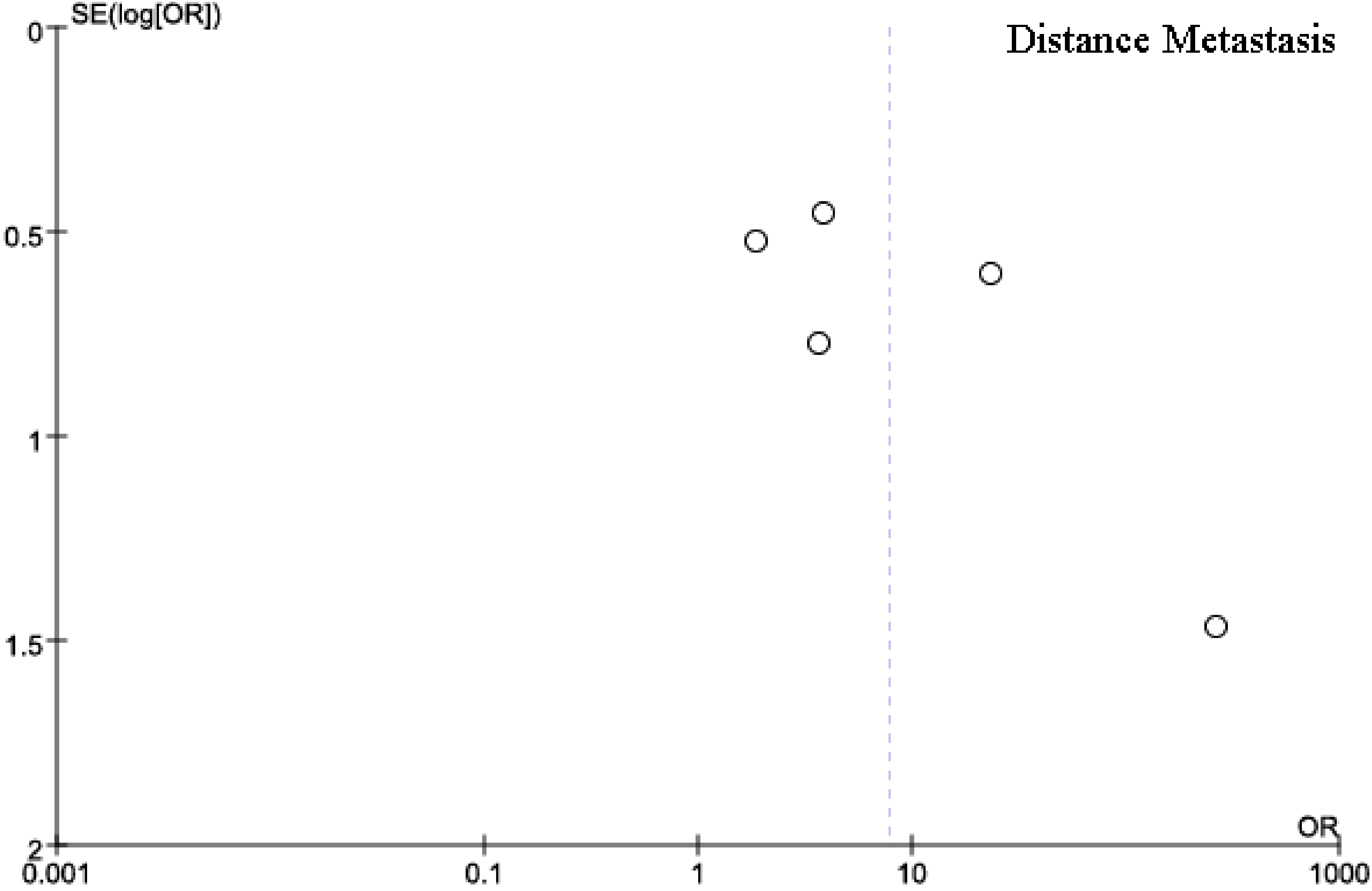
Distance Metastasis
SE(log[OR])
0
0.5
1
1.5
2
OR
0.001
0.1
1
10
1000

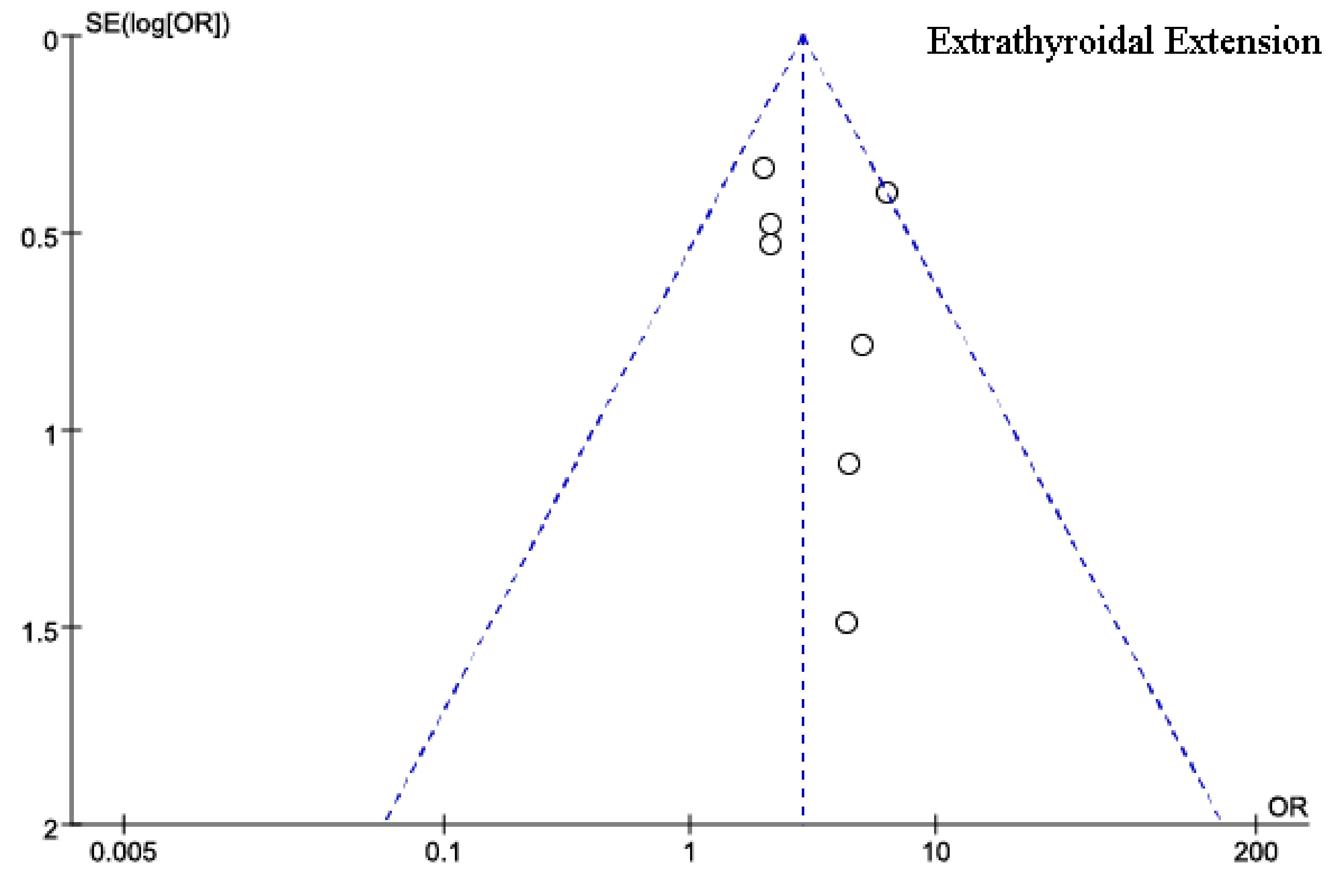

Extrathyroidal Extension
SE(log[OR])
0
0.5
1
1.5
2
OR
0.005
0.1
1
10
200

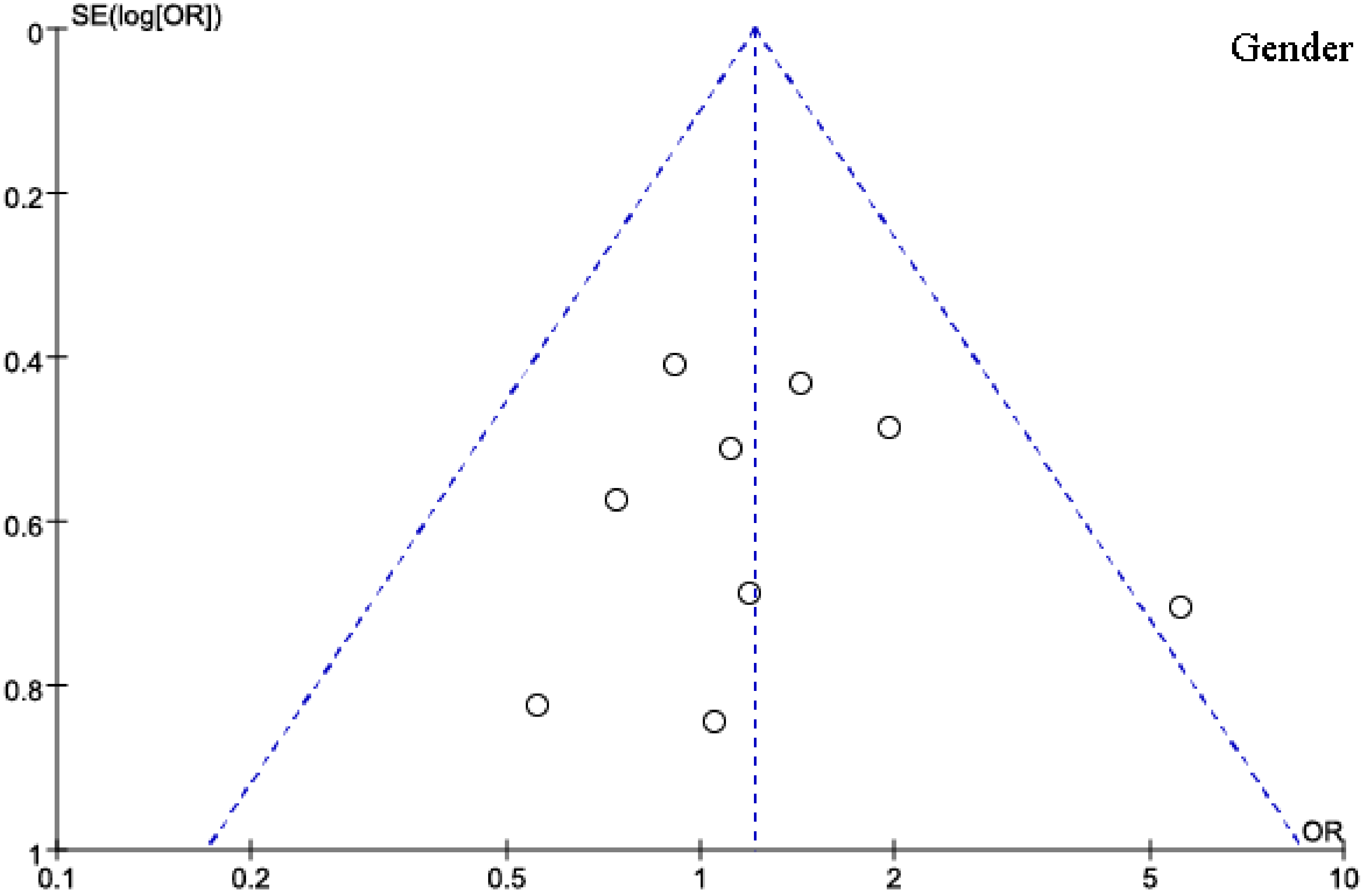
SE(log[OR])
Gender
0
0.2
0.4
0.6
0.8
1
OR
0.1
0.2
0.5
1
2
5
10

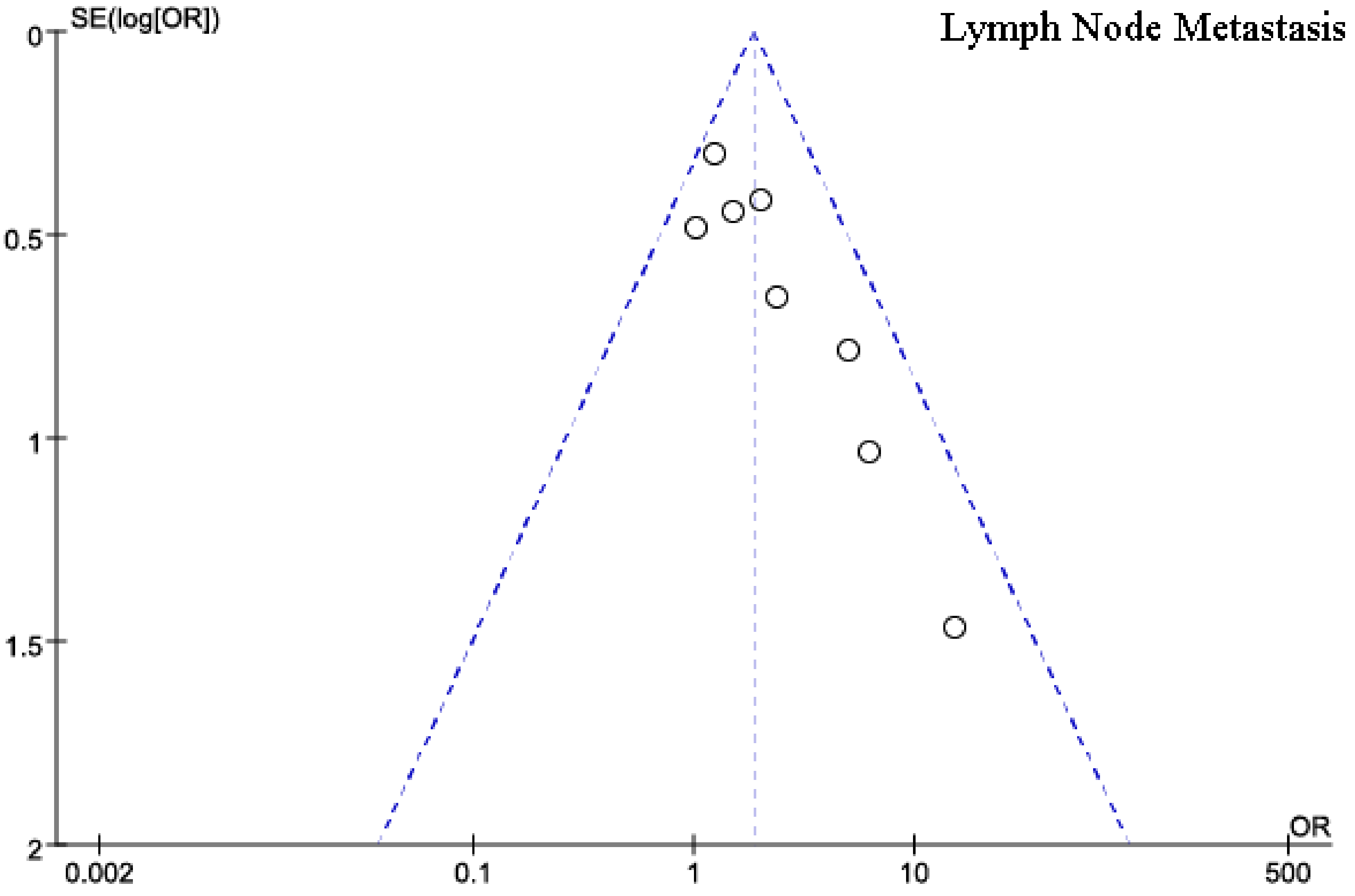

Lymph Node Metastasis
SE(log[OR])
0
0.5
1
1.5
2
OR
0.002
0.1
1
10
500

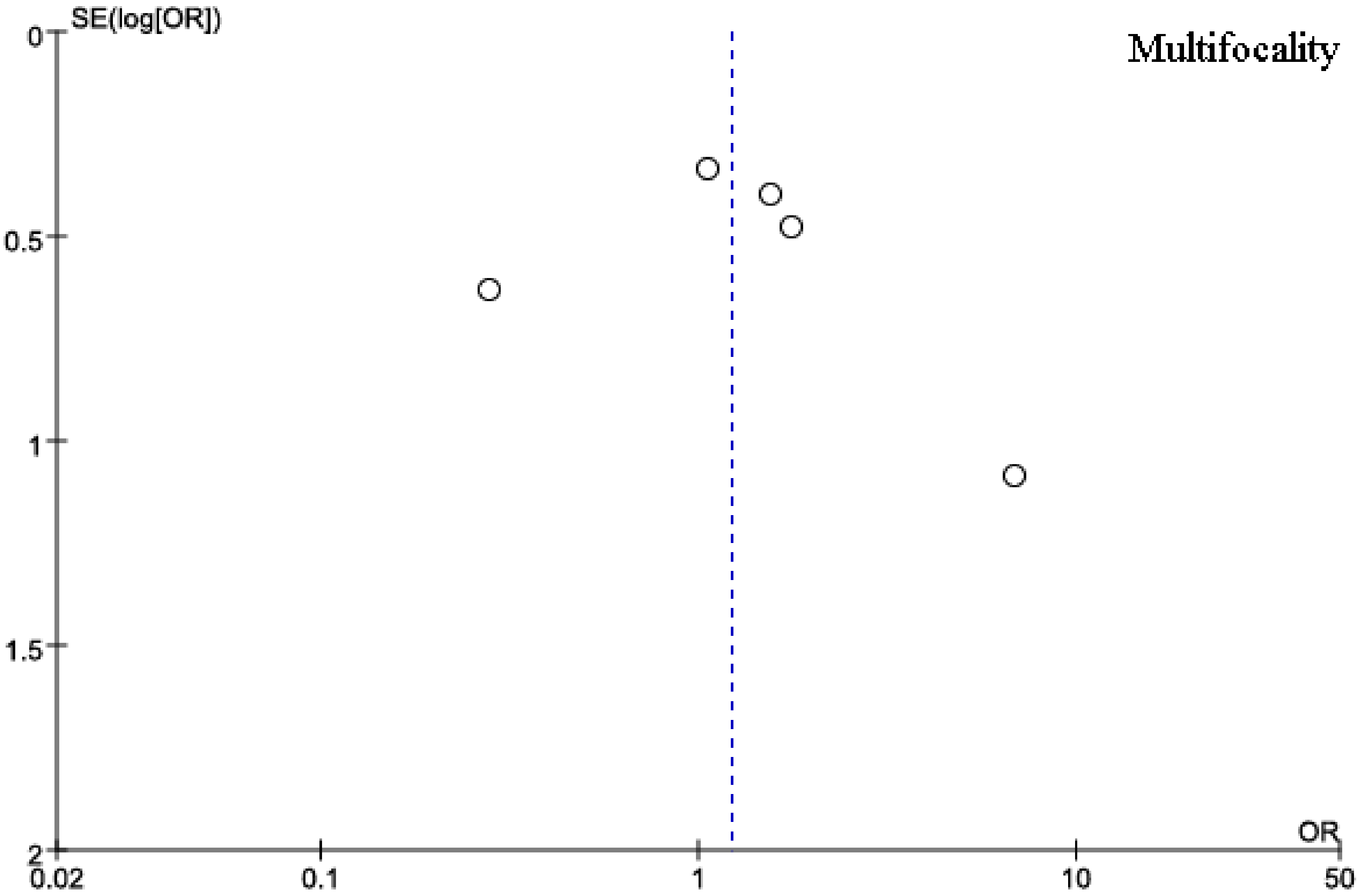

SE(log[OR])
Multifocality
0
0.5
1
1.5
2
OR
0.02
0.1
1
10
50

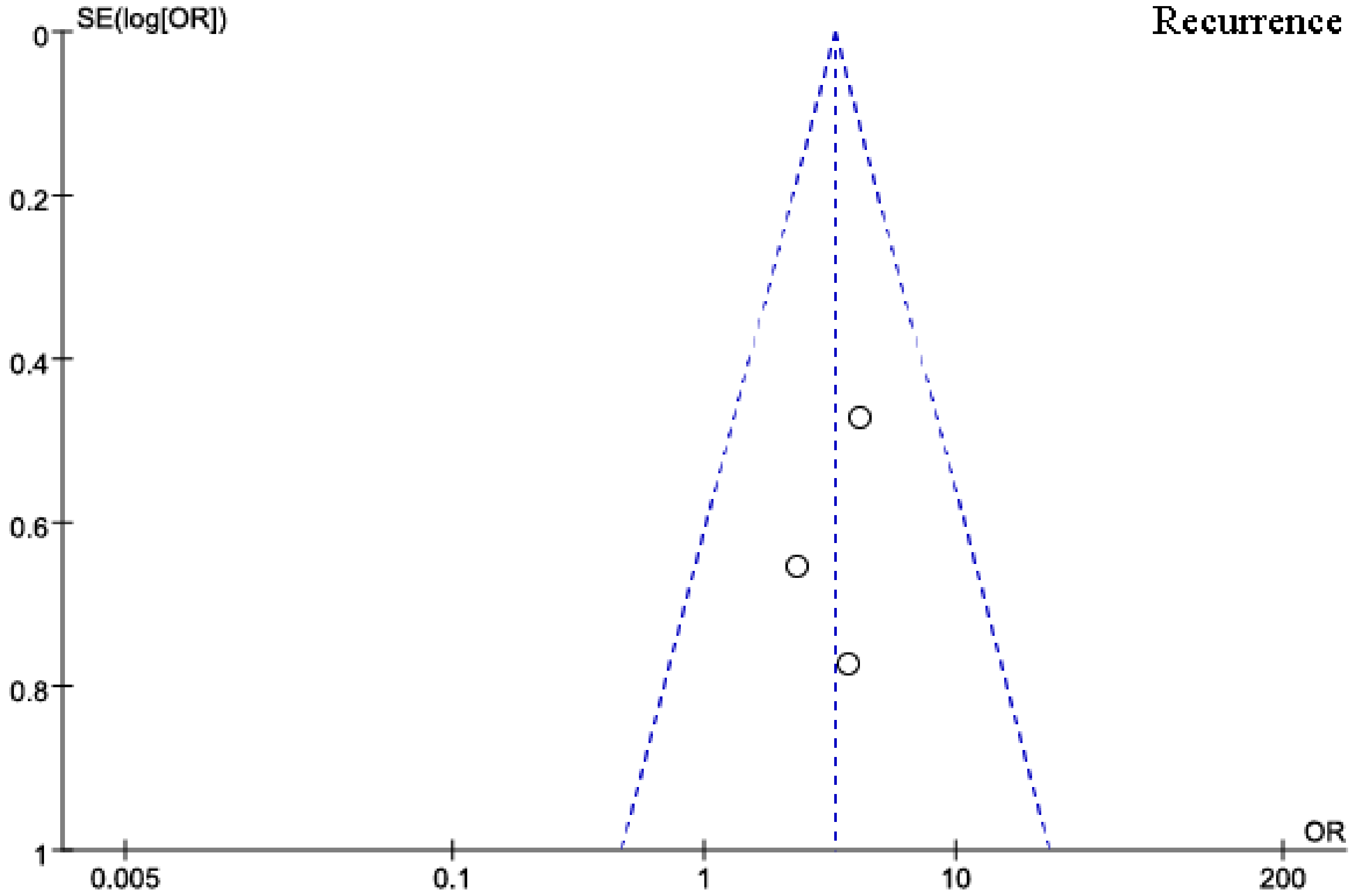
SE(log[OR])
Recurrence
0
0.2
0.4
0.6
0.8
1
OR
0.005
0.1
1
10
200

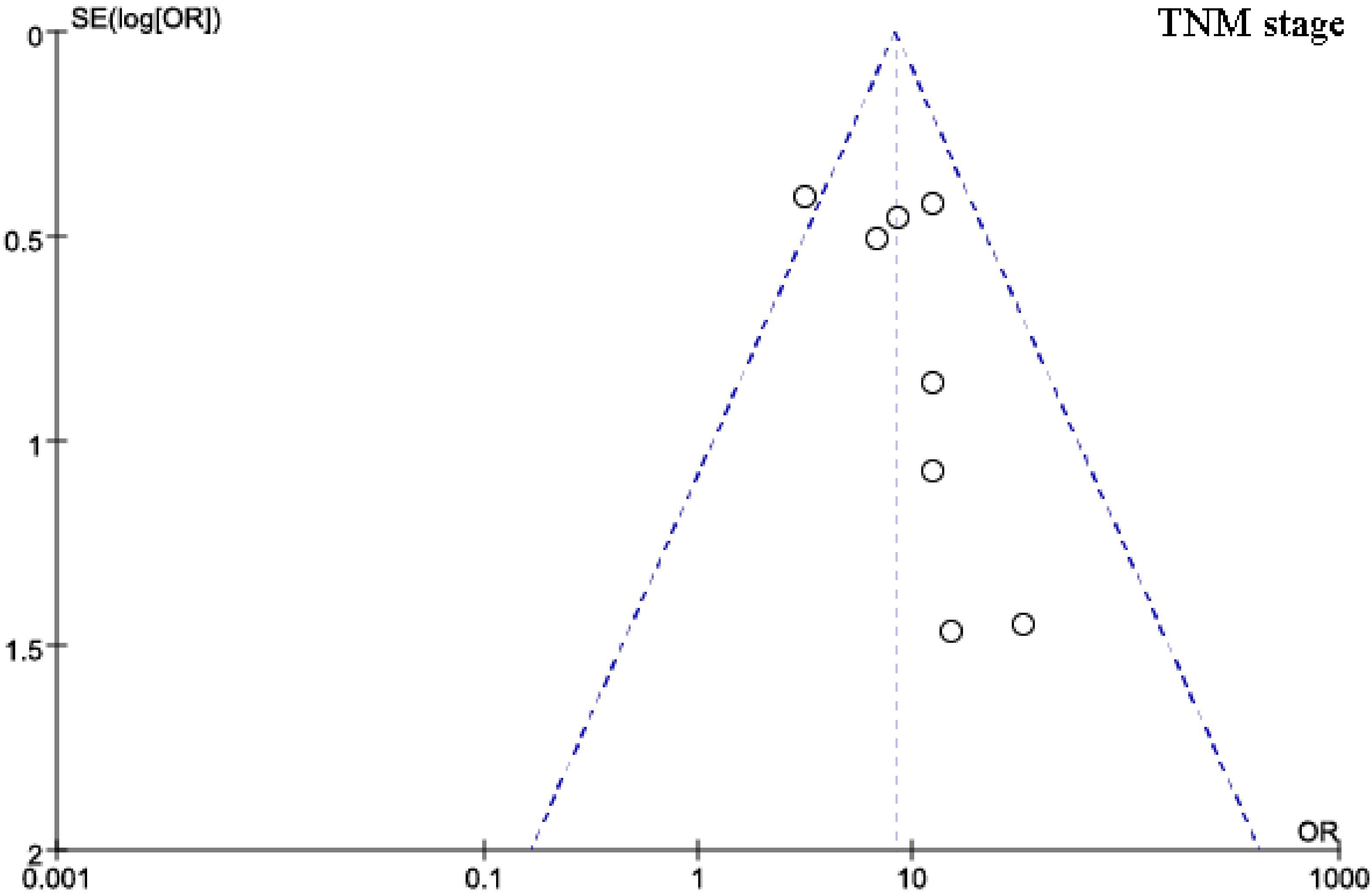

TNM stage
SE(log[OR])
0
0.5
1
1.5
2
OR
0.001
0.1
1
10
1000

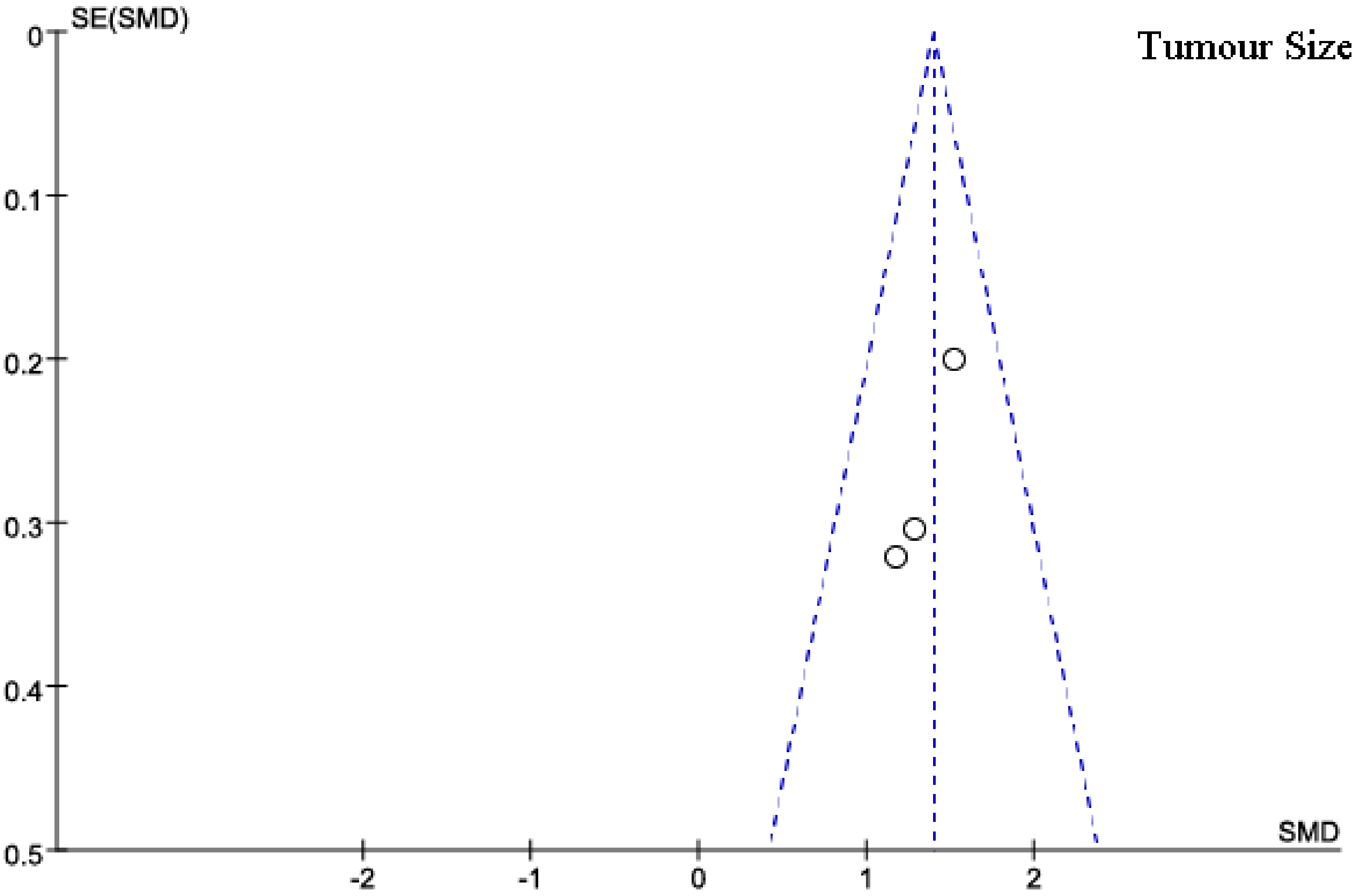

SE(SMD)
Tumour Size
0
0.1
0.2
0.3
0.4
0.5
SMD
-2
-1
0
1
2
